# OA27 Growth of the UK and Ireland paediatric rheumatology nurses’ group

**DOI:** 10.1093/rap/rkac066.027

**Published:** 2022-09-28

**Authors:** Heather Rostron, Polly Livermore

**Affiliations:** Leeds Teaching Hospitals NHS Trust, Leeds, United Kingdom; Great Ormond Street Hospital for Children NHS Foundation Trust Hospital and NIHR BRC, London, United Kingdom

## Abstract

**Introduction/Background:**

The Paediatric Rheumatology Clinical Nurse Specialist often has to manage a large caseload of children and young people. Paediatric Rheumatology is an umbrella term of over 80 conditions, most of which are long-term chronic illnesses which can be challenging for families to manage. The Clinical Nurse Specialist is therefore the first point of contact for families who want answers and guidance in caring for their child/young person. The UK and Ireland Paediatric Rheumatology Nurses Group, in turn, provides peer support to these nurses. This abstract will present the growth of this network, particularly over the last two years.

**Description/Method:**

Over two decades ago, a UK Paediatric Rheumatology Nurses group was established. Since the group’s formation, membership has grown from 20 to over 100 nurses, and has expanded into the Republic of Ireland. All nurses work in paediatrics and most are working solely in Rheumatology as Clinical Nurse Specialists (various titles exist). However, the group also contains nurses who may not solely focus on Rheumatology, but who also manage a number of specialities (one being Rheumatology), and those who have developed their own specialist interest in Rheumatology, often derived from providing clinical support to weekly Rheumatology clinics.  The group’s Lead Nurse has also encouraged Clinical Research Nurses supporting Paediatric Rheumatology studies to join, as the shared learning is useful to support their clinical practice too. Currently we only have one Paediatric Rheumatology Senior Clinical Research Nurse, but we do have some nurses who manage Paediatric Rheumatology studies as part of their wider clinical roles.

Members are located across 37 different centres in the UK and Ireland. Four of these centres have joined in the last month, with nurses hearing about the group and approaching the steering committee about their participation. The centres range from district general hospitals through to specialist regional Children’s hospitals. The seniority of our members ranges from band 5 through to band 8b, with three members managing Rheumatology services in a matron capacity. In fifteen of these centres, there is only one Paediatric Rheumatology Nurse within that centre, which can be isolating. The UK and Ireland group is accessible through email and WhatsApp and is always available for a quick question or check in. Keeping membership up to date, particularly with some nurses only joining for short periods of time to cover maternity leave, can be challenging. Tomorrow the numbers may have changed again!

**Discussion/Results:**

The growth of this group, particularly over the last two years, could be for a number of reasons:

1. Regular virtual meetings have been advertised on social media channels, especially via the British Society of Rheumatology (BSR). These don’t have to be sighted by Rheumatology nurses themselves but may have been noticed from other Rheumatology multidisciplinary team members, who then encouraged their nurses to make contact.

2. Having regular virtual meetings ensures that all of the Paediatric Rheumatology Nurses are invited and can take turns in attending and sharing best practice, so it is in a team’s best interest to encourage more hesitant nurses to ask to join.

3. The development of the WhatsApp group has provided quick and instantaneous responses and has clearly proven beneficial according to member feedback.

4. During the Covid-19 pandemic, working patterns changed with nurses being allowed to work from home. This change contributed to nurses feeling isolated from their peers, and also not having the wider multi-disciplinary team easily on hand and therefore asked the group their clinical questions.

5. Some members of the group have taken on additional roles, either within BSR or the Royal College of Nursing and this offers wider communication channels and increased visibility of the group through advertising.

6. Membership growth appears to mirror the growth seen in Rheumatology services, for example some centres have appointed Uveitis Clinical Nurse Specialist posts to work in conjunction with the Rheumatology Nursing Team.

7. The change in societal ways of working, with more work and meetings occurring virtually, and outside of the 9-5 office hours, means that nurses can attend meetings easier than having to expend time and finances to travel to face-to-face meetings.

8. New members joining naturally increases word of mouth and the wider reach of the group.

**Key learning points/Conclusion:**

Raising and maintaining the profile of this group is important. We know that there is no similar group for adult Rheumatology Nurse Specialists in the UK. Also, there is no other similar European Paediatric Rheumatology Nurses group. Paediatric Rheumatology is a huge speciality with nurses needing to be able to support families in their management of conditions outside of hospital appointments to prevent hospital admissions. The scope of the Rheumatology nurse is also always increasing, with pressure on nurses to undertake postgraduate studies, become nurse prescribers, carry out joint examinations, deliver nurse-led clinics and manage patients on immunomodulatory therapies in the community. The Paediatric Rheumatology Nurse also requires knowledge and skills in best practices for young people transitioning into adult services and be an expert in child development stages and the implications of these, whilst managing the needs and expectations of the child’s main carer and wider family. For these reasons alone, it is vital that we protect the Paediatric Rheumatology Nurse Specialist and ensure that they are supported, developed and valued, and therefore, stay in Rheumatology.

The ask of the wider multidisciplinary team is to allow Paediatric Rheumatology Nurses time to attend group meetings, encourage them to ask questions of the wider nursing group and to promote the group to new nurses or those who may not be aware of the group, to reach out and seek expert peer support.

Please contact: Dr Polly Livermore (PhD, MSc, BSc, Dip HE Nurse), Paediatric and Adolescent Rheumatology Nurses Lead for the UK and Ireland, if you would like to join this national group: polly.livermore@gosh.nhs.uk

